# Bending the rules: A novel approach to laryngeal surgery in a body donor study

**DOI:** 10.1002/hed.27939

**Published:** 2024-09-26

**Authors:** Linus L. Kienle, Leon R. Schild, Felix Boehm, Viola D. Hahn, Jens Greve, Adrian von Witzleben, Thomas K. Hoffmann, Patrick J. Schuler

**Affiliations:** ^1^ Department of Otorhinolaryngology – Head and Neck Surgery Ulm University Medical Center Ulm Germany; ^2^ Surgical Oncology Ulm i2SOUL Consortium Ulm Germany; ^3^ Department of Oto‐Rhino‐Laryngology University Hospital Heidelberg Heidelberg Germany

**Keywords:** 3D‐printing, difficult laryngeal exposure, laryngeal cancer, laryngeal surgery, transoral surgery

## Abstract

**Background:**

Transoral laser microsurgery, the standard surgical approach for early‐stage laryngeal cancer, necessitates an unobstructed line of sight to the operating field. However, achieving adequate laryngeal exposure can be challenging, potentially compromising treatment outcomes.

**Methods:**

We developed a 3D‐printed curved laryngoscope (sMAC), designed to match the upper airway anatomy. In a user study (*n* = 15) with a human body donor we compared the sMAC system to conventional microlaryngoscopy regarding laryngeal exposure and accessibility in a difficult exposure scenario.

**Results:**

All 15 participants achieved complete glottic exposure and successfully manipulated laryngeal landmarks using the sMAC system. Only four participants achieved partial exposure using microlaryngoscopy. Positioning of the sMAC system was significantly faster (*p* = 0.023). A vocal cord resection was conducted successfully (*n* = 2) using the sMAC system.

**Conclusion:**

The sMAC system effectively addresses challenges associated with transoral laryngeal surgery. Ongoing development aims to overcome current limitations of the system and prepare first clinical trials.

## 1 INTRODUCTION

Despite slowly declining incidence globally, laryngeal cancer still ranks among the most prevalent tumors occurring within the head and neck region. In 2020, approximately 184 615 new cases of laryngeal cancer were diagnosed worldwide, with 99 840 fatalities attributed to the disease.^1^ The treatment landscape for laryngeal cancer encompasses both radiation‐based and surgical strategies. Notably, transoral laser microsurgery (TLM) has emerged as the favored intervention for early‐stage laryngeal cancer (T1‐T2) in recent decades.^2^, ^3^ Compared to open partial laryngectomy, TLM offers shorter hospital stays and a faster return to normal swallowing function, while delivering comparable oncological outcomes.^4^, ^5^, ^6^ Its advantages over radiotherapy in treating early glottic cancer include a shorter overall treatment duration and higher rates of long term laryngeal preservation.^7^, ^8^

Nevertheless, TLM has a notable limitation. It relies on a laser beam, which necessitates an unobstructed line of sight through the oral‐oropharyngeal passage to access the surgical field effectively. This makes adequate exposure and visualization of laryngeal structures crucial.^9^, ^10^ However, in about 10%–20% of patients proper laryngeal exposure proves to be challenging, due to restricted cervical spine mobility, trismus, or extensive neck tissue scarring from prior radiation therapy.^11^, ^12^, ^13^ The indication of TLM for tumors involving the anterior commissure also poses difficulties as achieving suitable exposure of this region is especially challenging, potentially resulting in incomplete tumor removal and suboptimal oncological outcomes.^14^, ^15^ Additionally, the rigid operating laryngoscope employed in TLM procedures exerts considerable forces on the maxillary incisors and laryngopharynx, which may lead to postoperative complications in a significant proportion of patients, including transient laryngeal edema, hematoma, hypoglossal palsy, taste alterations, dysphagia, or dental injuries.^16^, ^17^, ^18^ To address these issues and provide surgical solutions for patients with difficult laryngeal exposure, it is essential to adapt surgical systems to the nonlinear anatomy of the laryngopharyngeal region.

In response to these challenges, our research group has introduced the sMAC system for laryngeal surgery. This system incorporates a curved video‐laryngoscope equipped with flexible instruments, allowing nonlinear access to the surgical site. Our previous research has demonstrated the system's ability to visualize and manipulate laryngeal structures in porcine larynx models and human body donor studies.^19^, ^20^ Additionally, our preclinical experiments confirmed that the sMAC system applies significantly less force to the upper front teeth and laryngopharynx as compared to direct rigid laryngoscopy used in TLM.^21^ However, it is important to note that the video laryngoscope was originally designed for intubation procedures and not for surgical applications. Its limitations include subpar image quality and a lack of zoom capabilities as compared to other available endoscopes and operating microscopes.

To address these limitations, we have developed an advanced iteration of the sMAC system. This new system is based on a 3D‐printed curved laryngoscope with integrated working channels. These channels accommodate a flexible video‐endoscope for real‐time visualization of the surgical field as well as flexible instruments for manipulation. We have previously evaluated the sMAC system regarding visualization and manipulation of laryngeal structures in an intubation dummy.^22^ The user study presented in this article aims to assess the enhanced system's capabilities in visualizing and manipulating laryngeal structures within a more realistic surgical setting and comparing it to conventional laryngoscopy in a difficult laryngeal exposure scenario. Additionally, we demonstrate a vocal cord resection using a nonlinear monopolar needle instrument in a human body donor.

## 2 MATERIALS AND METHODS

### 2.1 Design characteristics of the sMAC laryngoscope

**FIGURE 1:**
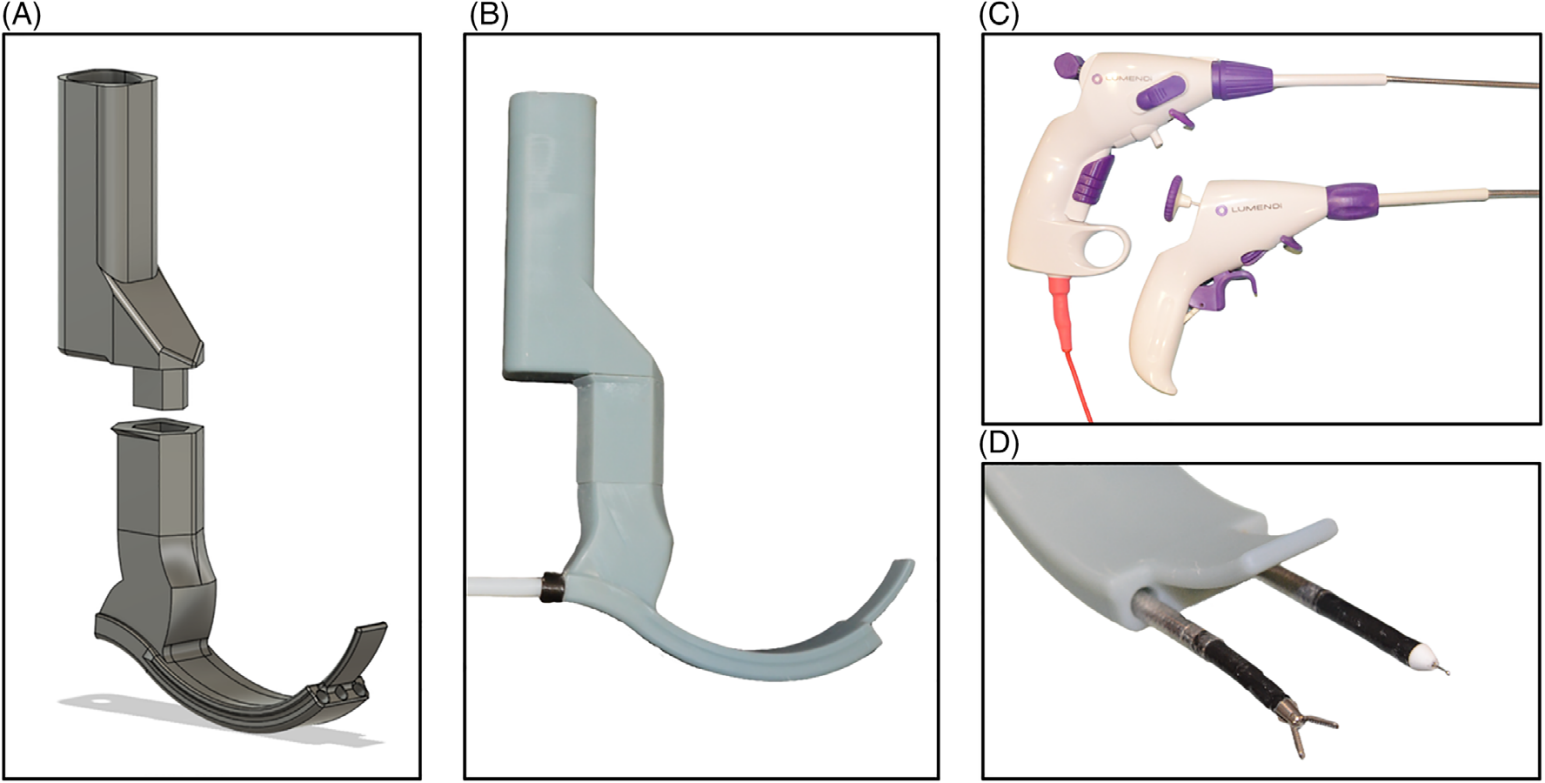
(A) CAD model of the sMAC laryngoscope. (B) Fully 3D‐printed and assembled sMAC laryngoscope. (C) Control units of monopolar needle instrument (above) and the grasper instrument (below). (D) Tips of the fully flexible surgical instruments inserted into the working channels. [Color figure can be viewed at wileyonlinelibrary.com]

The design of the sMAC laryngoscope (Figure 1A,B) is characterized by its hyper‐angulated configuration tailored to the complex nonlinear anatomy of the upper aerodigestive tract. This specialized demonstrator incorporates three working channels with a 6.5‐mm diameter, intended to enable instrument access to the surgical site and accommodate a flexible video endoscope. Located at the distal end of the demonstrator a spatula holds the epiglottis in place, preventing it from obstructing the surgical field's visibility. In contrast to the previous version of the sMAC system, this advanced iteration now integrates a detachable mount for the flexible endoscope into the handle of the prototype. The prototype was manufactured using an LCD printing process with an LD‐002H 3D printer (Creality, Shenzen, China). Because the size of the prototype exceeded the printer bed size, the blade and grip of the laryngoscope were printed separately and assembled afterwards. To ensure the prototype's durability during surgical use, a specialized high‐strength engineering resin was utilized for printing (Build Resin, Siraya Tech, San Gabriel, CA).

### 2.2 Flexible video endoscope

For visualizing the surgical field, we employed a flexible video endoscope (11101HDK, Karl Storz, Tuttlingen, Germany). The endoscope has a 30‐cm working length and a 3.7‐mm outer diameter. It features a high‐definition camera at its tip with a 100° field of view, and the endoscope tip allows deflection of up to 140°. The endoscope's images were displayed on an 18.5‐inch TP101 monitor from Karl Storz with a resolution of 1920 × 1080 pixels.

### 2.3 Instrumentation

Facilitating access through the curved pathways of the working channels necessitated the utilization of fully flexible instruments. We adopted instruments from the DiLumen C2 system (Lumendi, Maidenhead, UK), as depicted in Figure 1C,D. These manually operated, single‐use instruments possess an outer diameter of 6 mm. The DiLumen Ig grasper features 6 mm long jaws with a 60° opening angle. The grasper's tip is capable of 90° deflection in two directions, providing a hemispherical working space. In addition to the grasper, the DiLumen Ik monopolar electrosurgical knife from Lumendi was used in our experiments. This instrument has a 4‐mm long extendable blade and allows 90° deflection of the instrument tip in one direction. All instruments were shortened to a length of 55 cm to suit our transoral laryngeal approach.

### 2.4 Experimental setup

For our experiments a fresh‐frozen human body donor was placed on the operating table. To simulate difficult laryngeal exposure, a cervical support collar was applied, which restricted the mobility of the cervical spine as well as opening of the mouth. Ethical approval for experiments involving human body donors was obtained from the local ethics committee (# 89/19).

**FIGURE 2:**
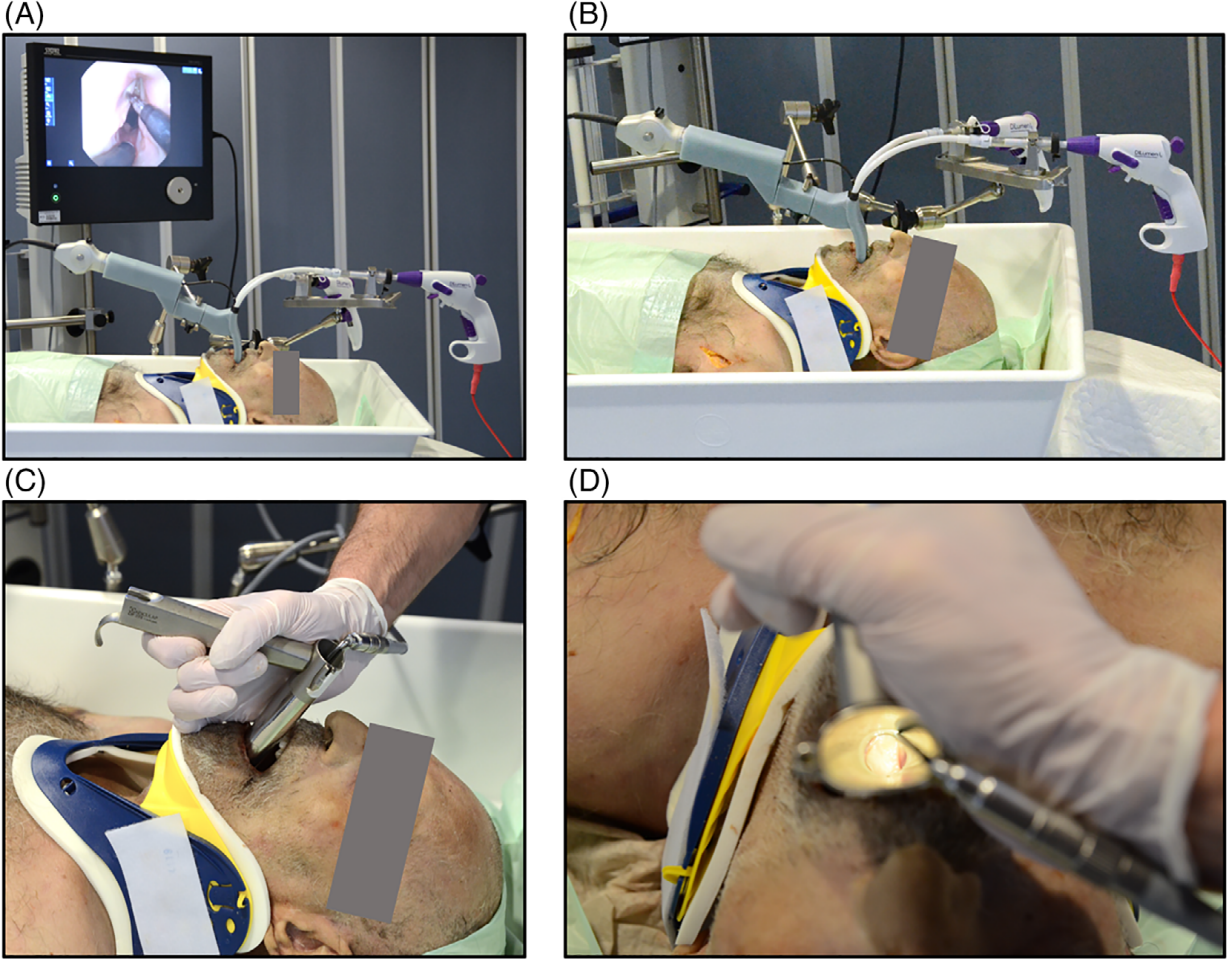
Experimental setup of body donor study. The mobility of the cervical spine and mouth opening was restricted by a cervical support collar. (A, B) The sMAC laryngoscope was attached to the operating table using an articulated stand. The flexible video endoscope was placed into the integrated mount and the endoscopic image displayed on an 18.5‐inch Monitor. (C, D) Kleinsasser operating laryngoscope equipped with a fiberoptic light carrier. [Color figure can be viewed at wileyonlinelibrary.com]

The 3D‐printed sMAC laryngoscope was secured to the operating table using an articulated stand (28272 HA, Karl Storz) in conjunction with a clamping jaw (28272 UFN, Karl Storz). A metal instrument holder (Lumendi) positioned at the head of the operating table supported the proximal ends of the instruments, enabling simultaneous use of both instruments. The flexible endoscope was inserted into the central working channel for visualization and placed into the mount attached to the handle of the sMAC laryngoscope. The demonstrator was then inserted into the pharynx of the body donor using the flexible endoscope as a visual guide. After ensuring proper positioning and full exposure of the larynx, the articulated stand was secured by fastening its central mechanical clamp (Figure 2A,B). For comparison, a Kleinsasser operating laryngoscope (OP292, Aesculap, Tuttlingen, Germany) commonly used in TLM was selected, which was additionally equipped with a fiberoptic light carrier (Figure 2C,D).

### 2.5 User study

We conducted a comparative analysis to assess the sMAC system's efficacy in visualizing laryngeal structures in a patient exhibiting cervical spine mobility limitations, in comparison to conventional direct laryngoscopy. The study encompassed 15 participants, comprising five specialist practitioners routinely engaged in TLM procedures, along with 10 residents from our Otorhinolaryngology (ENT) department.

Initially, participants were tasked with positioning the operating laryngoscope in order to expose the glottic plane of the body donor. Subsequently, we recorded the time to achieve proper laryngoscope placement. Additionally, a photographic record of the observable regions within the glottic plane was captured through the laryngoscope's aperture following completed positioning.

In the second phase of the experiment, participants were instructed to position the sMAC laryngoscope and visualize the glottic plane within the body donor. Once again, the time elapsed to position the sMAC laryngoscope was documented, and an image of the exposed glottic plane was acquired.

After positioning the sMAC system the participants were asked to reach for the following laryngeal landmarks using the grasper instrument: Left and right vocal folds, left and right vestibular fold, anterior commissure, postcricoid region, subglottic region. The time to complete this task again was documented.

### 2.6 Vocal cord resection

Using the identical experimental configuration with an additional endotracheal tube placed in the trachea of a body donor, we conducted a vocal cord resection procedure employing the sMAC system. The vocal cord resection was carried out by a proficient specialist in the field of laryngeal surgery. For manipulation and handling of the vocal cords, the DilLumen grasper instrument was employed, while cutting operations were executed using the DiLumen monopolar needle instrument. The VIO 300D electrosurgical generator (Erbe Elektromedizin GmbH, Tübingen, Germany) was used to power the monopolar needle instrument. After several trials we found the auto cut setting with an effect size of two and a maximum power output of 30 W most suitable to perform in laryngeal tissue. Smoke from cauterized tissue was removed by a suction line strategically placed in the pharyngeal region directly above the surgical field.

### 2.7 Statistics

Statistical analysis was conducted with MATLAB and the included statistics and machine learning toolbox (MathWorks, Natick, MA). The measured times were checked for normality using the Shapiro–Wilk test. To compare the times needed to position the sMAC system to the setup time of the laryngoscope, a paired sample *t*‐test was performed. The differences between the group of residents as compared to the group of specialists were evaluated using a two‐sample *t*‐test. Statistical significance was considered for *p*‐values <0.05. Results are given in the form mean ± standard deviation unless stated otherwise.

## 3 RESULTS

**FIGURE 3:**
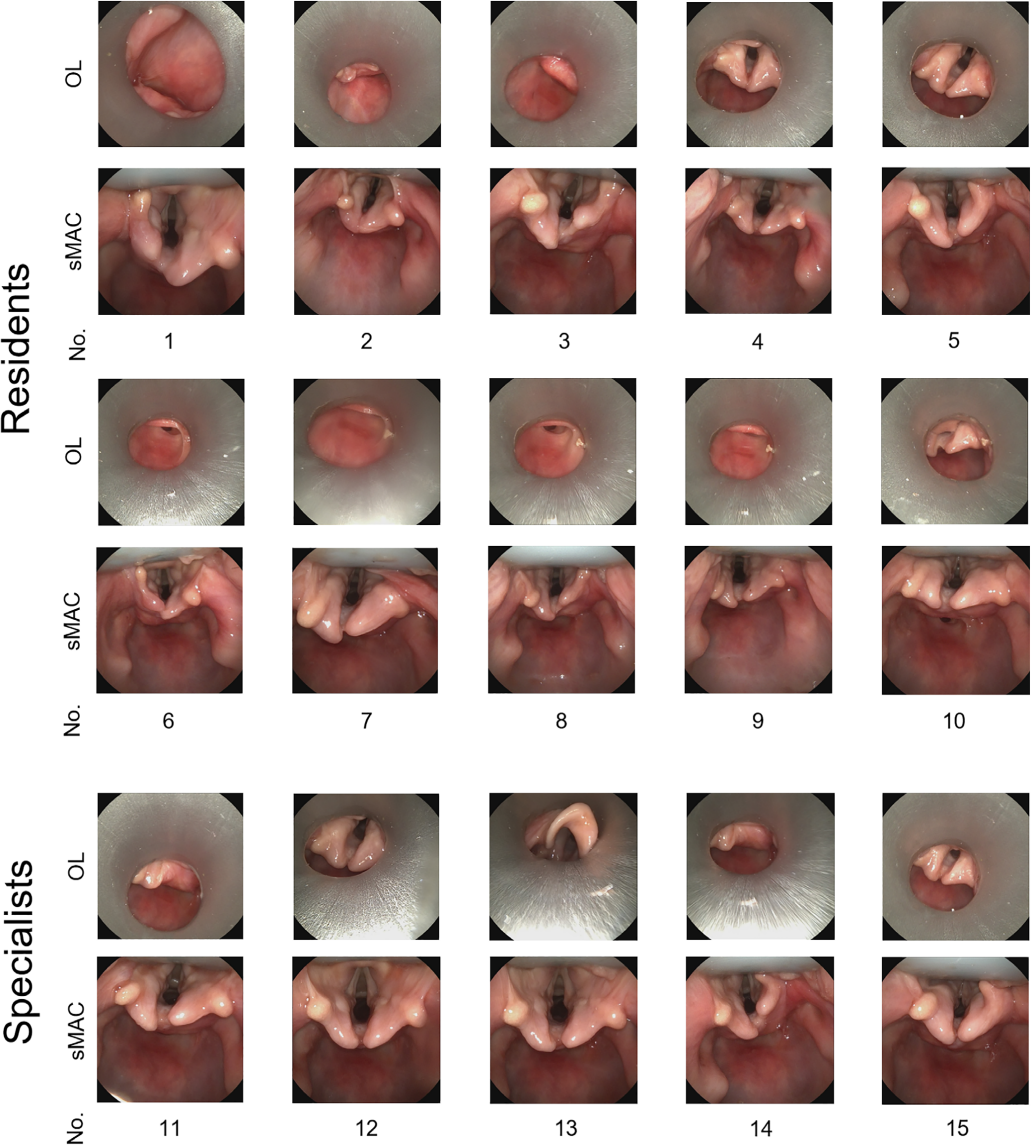
Photographic record of the user study. Endoscopic images of the final position of the sMAC laryngoscope (sMAC) and Kleinsasser operating laryngoscope (OL), respectively, for each participant of the user study. Only study participant No. 4, 5, 12, and 15 were able to expose the posterior parts of the glottic plane using the operating laryngoscope. In contrast all participants successfully visualized the entire glottic plane employing the sMAC system. [Color figure can be viewed at wileyonlinelibrary.com]

Figure 3 illustrates the acquired images from the user study, demonstrating the placement of both the sMAC system and the Kleinsasser laryngoscope for each participant in the study. Out of the 10 residents involved in the study, only two managed to partially expose the glottic plane when utilizing the conventional operating laryngoscope. In contrast, all 10 residents successfully visualized the glottic plane of the body donor when employing the sMAC system.

Similarly, among the five ENT specialists who participated, only two were able to position the conventional operating laryngoscope in a manner that exposed the glottic plane, while all of them achieved complete visualization of the glottic plane when utilizing the sMAC system.

**FIGURE 4:**
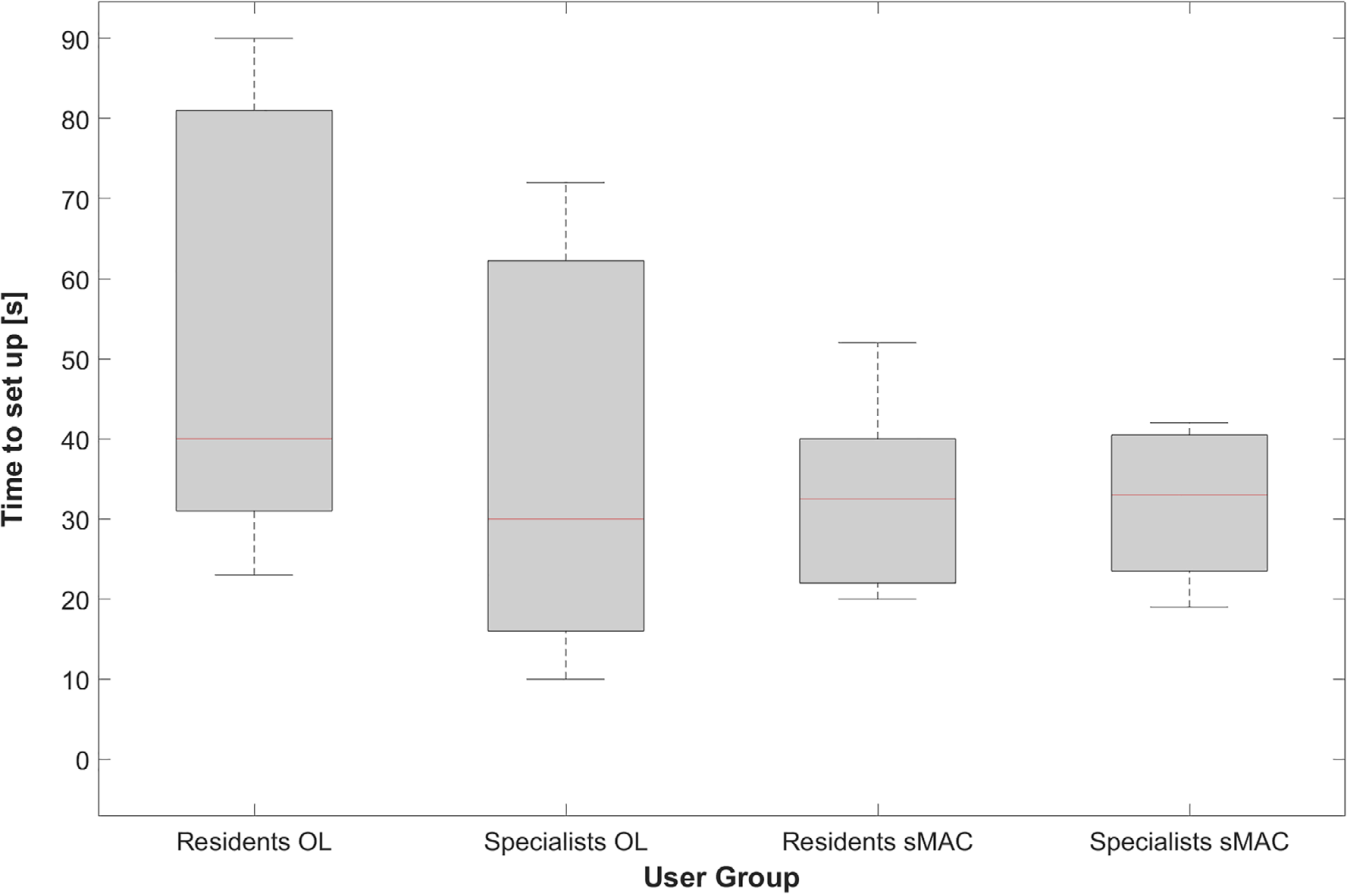
Boxplot of the time required for setting up the operating laryngoscope as compared to the sMAC system within each user group. [Color figure can be viewed at wileyonlinelibrary.com]

Figure 4 displays a boxplot illustrating the time required for setting up the operating laryngoscope as compared to the sMAC system within each user group. The participants needed significantly less time to position the sMAC system as compared to the conventional Kleinsasser laryngoscope (32.7 s ± 10.4 s vs. 47.3 s ± 26.9 s, *p* = 0.023). ENT specialists exhibited faster positioning times for the Kleinsasser laryngoscope as compared to residents (37.8 s ± 26.7 s vs. 52.1 s ± 27.1 s, *p* = 0.175). However, this result showed no statistical significance. Additionally, there was no statistically significant difference in the time required to position the sMAC system (33.1 s ± 11.2 s vs. 31.8 s ± 9.8 s, *p* = 0.882).

**FIGURE 5:**
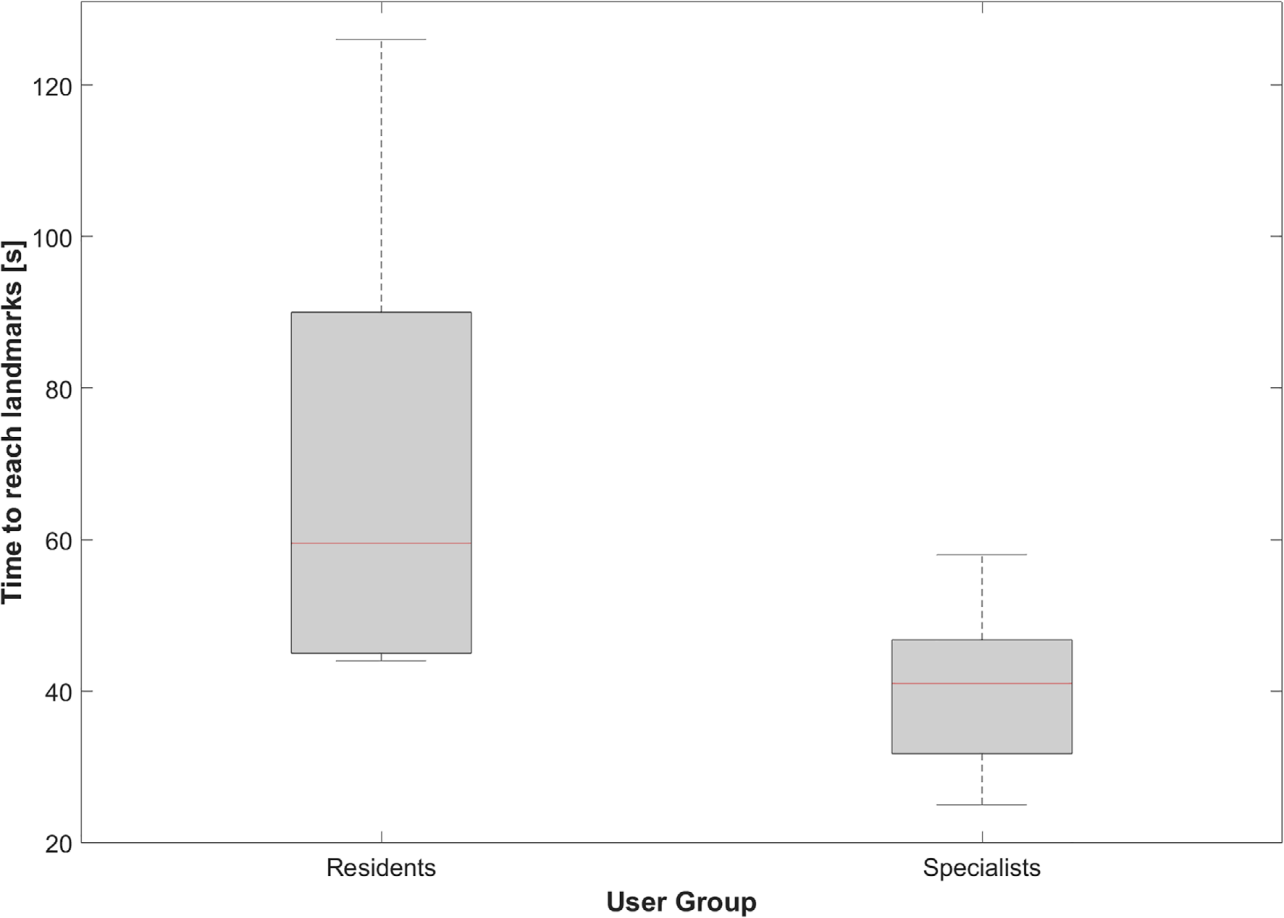
Boxplot of the time required to reach for the predefined laryngeal landmarks with the grasper instrument using the sMAC system for each user group. [Color figure can be viewed at wileyonlinelibrary.com]

All study participants successfully reached the predefined laryngeal landmarks with the grasper instrument using the sMAC system. Figure 5 provides a boxplot graph of the time necessary to accomplish this task. In this context, ENT specialists completed this task significantly faster than the residents (40.2 s ± 12.2 s vs. 70.0 s ± 29.1 s, *p* = 0.008).

**FIGURE 6:**
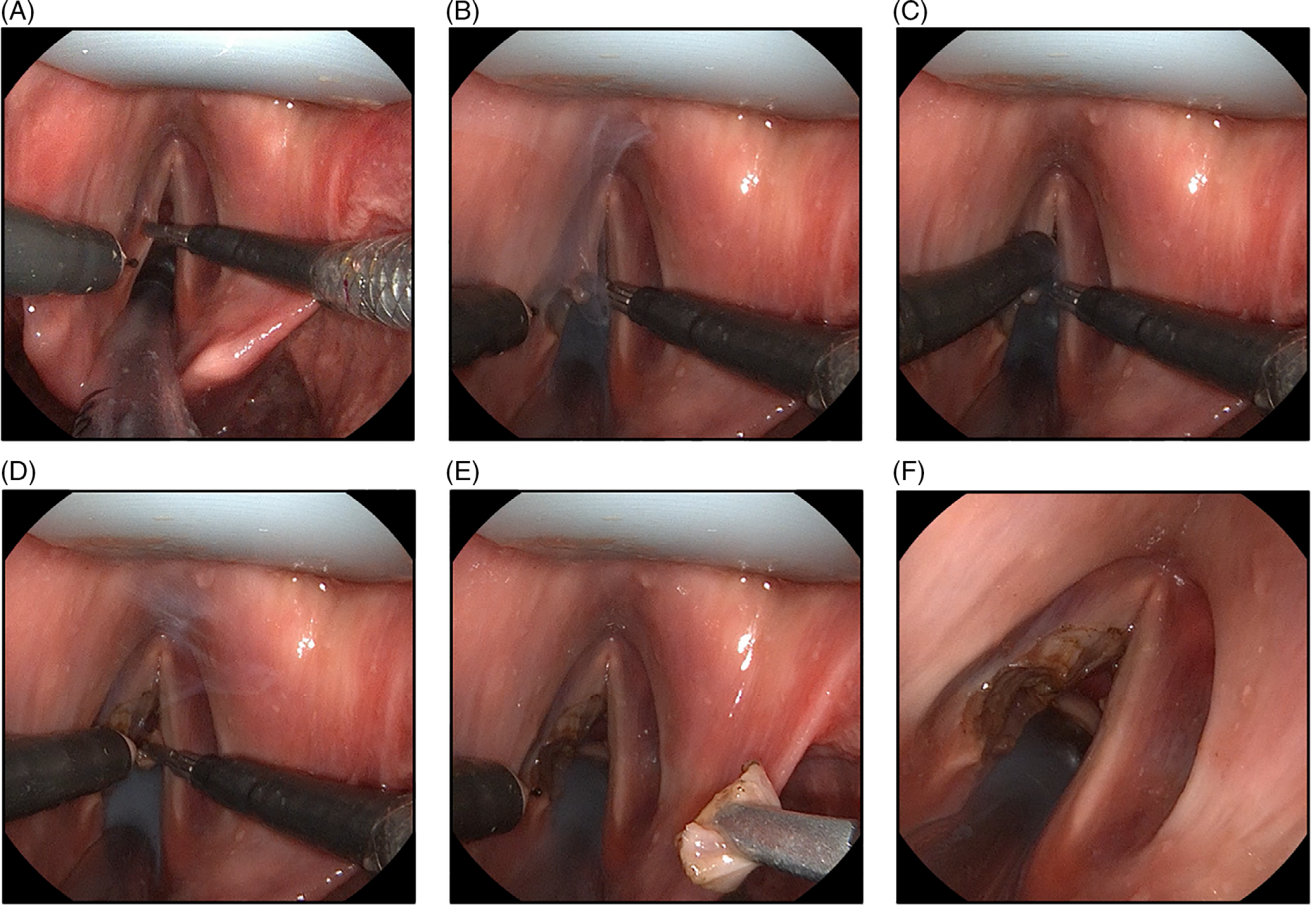
Resection of the left vocal fold of the body donor using the sMAC system. (A–D) Wedge‐shaped incisions using the DiLumen monopolar needle instrument while tensioning the target area with the grasper instrument. (E, F) Extraction of the removed tissue and close‐up view of the operating field. [Color figure can be viewed at wileyonlinelibrary.com]

In a subsequent experiment, we performed a resection of the left vocal fold on the body donor using the sMAC system, as detailed in Figure 6. The procedure involved employing the DiLumen grasper instrument to tension the vocal fold at the target area, followed by the execution of two wedge‐shaped incisions using the DiLumen monopolar needle instrument to remove the tissue surrounding the target area. The entire procedure was successfully completed in 4 min and 19 s. In a subsequent attempt, we performed a resection of the right vocal fold using the same approach, which took 4 min and 40 s.

## 4 DISCUSSION

TLM is widely regarded as the modern surgical approach for the treatment of early‐stage laryngeal cancer. It is a well‐established system with robust clinical evidence supporting its efficacy, even in comparison to alternative treatment modalities such as radiation therapy.^7^, ^8^, ^23^ However, TLM does have limitations. Particularly in cases involving lesions of the anterior commissure achieving adequate exposure can be challenging or even impossible, potentially resulting in suboptimal treatment outcomes.^14^, ^15^ Additionally, in such cases the operating laryngoscope exerts significant forces to the laryngopharynx and maxillary incisors, which may lead to increased tissue damage and subsequent postoperative complications.^16^, ^17^, ^18^

Studies suggest that 10%–20% of patients undergoing suspension laryngoscopy reveal difficult laryngeal exposure. Piazza et al. developed the laryngoscore to predict difficult laryngeal exposure. Part of the laryngoscore is the degree of neck flexion‐extension, thyreo‐mental distance and the interincisor gap, which heavily correlate with difficult laryngeal exposure.^11^, ^12^, ^13^

In our user study designed to simulate difficult laryngeal exposure, we restricted the mobility of the cervical spine of the human body donor using a cervical support collar. The collar limited the degree of neck flexion‐extension and reduced the interincisor gap as well as the thyreo‐mental distance. These limitations prevented participants from positioning the body donor in the Boyce‐Jackson position, which typically straightens the naturally curved oral‐oropharyngeal pathway without applying excessive force to oropharyngeal tissues. As a result, only 4 out of 15 participants were able to expose parts of the glottic plane using conventional laryngoscopy. However, the exposure was limited to the posterior one‐third or less of the glottic plane, which in our opinion cannot be considered adequate laryngeal exposure. Due to their experience, the specialist group achieved laryngeal exposure more frequently and in less time with conventional direct laryngoscopy. But none of the participants was able to expose the entire glottic plane including the anterior commissure.

In contrast, all participants succeeded in exposing the entire glottic plane using the sMAC system. Because of the curved shape of the sMAC system, the restricted mobility of the cervical spine did not compromise the systems' capability to visualize the glottic plane. Notably, since the sMAC system is a novel technology, neither residents nor specialists exhibited differences in the time required to position the sMAC system, as both groups were equally unfamiliar with its handling. These results suggest, that positioning of the sMAC system is user‐friendly and does not require special technical experience.

In our final experiment we successfully demonstrated a transmuscular vocal cord resection. The procedure was executed using the grasper and monopolar needle instrument. The grasper instrument allowed precise tissue manipulation and provided a firm grip. The monopolar needle instrument performed cuts effortlessly and with minimal thermal tissue damage along the incision site. The suction tube placed above the surgical field removed smoke from cauterized tissue fast enough to prevent impairment of vision and the endotracheal tube did not hinder access to the surgical field. Limitations of our user study include the small cohort size of 15 study participants and the availability of only one body donor to conduct our experiments on.

In the evaluation of the sMAC system for laryngeal surgery, several advantages and disadvantages have been identified. On the positive side, our user study showed that the sMAC system offers exposure of the vocal cords, even in cases where conventional laryngoscopy fails to achieve adequate exposure, providing a valuable solution for challenging surgical conditions. Moreover, as described before, the curved shape of the sMAC laryngoscope exerts less force on the oropharyngeal tissue, possibly reducing postoperative complications.^21^ Leveraging 3D printing for its components contributes to cost‐effectiveness and offers the potential for customization to match individual patient anatomy. To adapt the sMAC system to individual patient anatomy, the curvature of the patient's upper airway is initially measured using MRI or CT scan. In the CAD software environment, the curve of the sMAC system's blade is adjusted to match this measured curvature, resulting in the creation of a patient‐specific 3D model of the sMAC laryngoscope. This model can then be manufactured using 3D printing technology. The continuous flexible instruments provide some tactile feedback to the surgeon during tissue manipulation, and the flexible endoscope provides high‐quality images of the surgical field from various angles and distances. On the downside, the material used to print the sMAC prototype is not FDA‐approved, although there are similar materials with FDA‐approval commercially available. Additionally, the substantial investment in single‐use instruments contributes significantly to the overall costs incurred when employing the sMAC system. However, in cases where TLM is indicated but not feasible due to challenging laryngeal exposure, alternative treatments such as radiotherapy often incur higher costs as well.^24^, ^25^, ^26^ Under these circumstances, the proposed system may offer a financially viable alternative. While the monopolar needle performed satisfactory in our experiments, incorporation of laser technology into the sMAC system could further mitigate thermal injury and carbonization. Furthermore, the end effectors originally designed for colorectal surgery may require size reduction to accommodate the more intricate structure of the human larynx. Lastly, the sMAC system represents a novel approach, necessitating dedicated training for users to fully harness its potential.

In the last decade, transoral robotic surgery (TORS) emerged as a promising alternative to TLM. Currently, the DaVinci system from the company Intuitive Surgical is the predominant choice for performing robot‐assisted head and neck surgeries worldwide.^27^ In contrast to the sMAC system, the DaVinci system introduces a different set of pros and cons. On the positive side, the DaVinci system represents a well‐established platform across various surgical domains, offering a three‐dimensional view of the operative site and precise controls, which augment surgical precision. Furthermore, the use of wristed instruments may circumvent some line‐of‐sight limitations that are met in TLM.^28^ However, there are several drawbacks to consider. Although no direct line‐of‐sight is necessary, the requirement for a straight access path to insert the rigid instruments may still limit its utility. Exposing the entire glottic plane is not feasible in specific cases such as reduced mouth opening, limited mobility of the cervical spine, macroglossia, and retrognathia.^28^, ^29^, ^30^, ^31^

An approach that circumvents this limitation of transoral approaches using the DaVinci system is the robotic‐assisted extended “Sistrunk” approach (RESA). This technique accesses the larynx via a single submental incision, which also permits access to lateral neck lymphatics. Consequently, although being a more invasive procedure compared to transoral approaches, RESA may be particularly beneficial for patients with glottic or supraglottic lesions who require concurrent neck dissection.^32^, ^33^

A fundamental drawback of the DaVinci system is the absence of tactile feedback, which can be critical in surgical oncology.^34^, ^35^ The instrument limitations of the sMAC system also apply to the DaVinci system. The size of the instruments is not optimal for laryngeal surgery and laser technology instruments are not natively supported by the system.^28^ Moreover, the evidence for using DaVinci in glottic lesions is limited, with most studies focusing on supraglottic pathologies, suggesting that its applicability to glottic lesions may require further investigation and validation in large multicentric randomized clinical studies.^30^, ^36^, ^37^ Finally, the financial investment to conduct robotic surgery today remains still considerably higher as compared to TLM or open approaches.^38^

## 5 CONCLUSION

In conclusion, the sMAC system exhibits promise in addressing challenges associated with laryngeal surgery. Particularly in cases where achieving adequate laryngeal exposure is difficult or unfeasible and therefore not suitable for TLM or TORS, the system could present an alternative surgical treatment modality in the future. Nevertheless, it is essential to acknowledge the current limitations of the system that warrant further examination and refinement before its adoption in clinical practice. In this context our research group currently develops a prototype constructed from FDA‐approved materials in preparation for first clinical trials.

## AUTHOR CONTRIBUTIONS

LLK constructed the prototype, performed experiments, and wrote the paper. LRS and FB performed experiments. AW and VH analyzed results. JG designed research. TKH analyzed results. PJS designed research, performed experiments, and wrote the paper. All authors have revised and approved the manuscript.

## CONFLICT OF INTEREST STATEMENT

The authors declare no conflicts of interest.

## Data Availability

The data that support the findings of this study are available from the corresponding author upon reasonable request.
